# Urine-to-Serum Osmolality Ratio as a Prognostic Marker in Traumatic Brain Injury

**DOI:** 10.3390/diagnostics16071071

**Published:** 2026-04-02

**Authors:** Eun Young Kim, Jeong-Am Ryu

**Affiliations:** 1 Department of Critical Care Medicine, Samsung Medical Center, School of Medicine, Sungkyunkwan University, Seoul 06351, Republic of Korea; eunyoungari.kim@samsung.com; 2 Department of Neurosurgery, Samsung Medical Center, School of Medicine, Sungkyunkwan University, Seoul 06351, Republic of Korea

**Keywords:** craniocerebral trauma, osmolar concentration, prognosis, Glasgow Coma Scale, intensive care units, pituitary gland

## Abstract

**Background/Objectives**: Prognostication in traumatic brain injury (TBI) remains challenging. The urine-to-serum osmolality (U/S) ratio may reflect hypothalamic–pituitary axis integrity, a critical but underexplored prognostic domain. We investigated whether the U/S ratio provides early prognostic value and enhances prediction when combined with conventional severity markers. **Methods**: This retrospective study included 128 adult TBI patients admitted to a neurosurgical intensive care unit (ICU) with simultaneous osmolality measurements within 6 h of admission. The primary outcome was ICU mortality; the secondary outcome was poor neurological outcomes (Glasgow Outcome Scale 1–3). **Results**: ICU mortality was 14.1% (18/128), and poor neurological outcome occurred in 41.8% (46/110). Non-survivors had significantly lower U/S ratios than survivors (1.09 ± 0.58 vs. 1.70 ± 0.68, *p* < 0.001). For ICU mortality, U/S ratios (AUC = 0.803) showed similar discriminative ability to GCS (AUC = 0.806). For poor neurological outcomes, the U/S ratio (AUC = 0.768) significantly outperformed both GCS (AUC = 0.641, *p* = 0.038) and the Acute Physiology and Chronic Health Evaluation (APACHE) II score (AUC = 0.553, *p* < 0.001). Combining the U/S ratio with GCS improved mortality prediction (AUC = 0.890), as did combinations with the APACHE II score (AUC = 0.847). The U/S ratio remained independently associated with ICU mortality and poor neurological outcomes after adjusting for GCS or APACHE II scores. Quartile analyses revealed a dose–response relationship, with ICU mortality of 34.4% in Q1 versus 3.1% in Q4 (*p* for trend < 0.001). Prognostic value was preserved in patients receiving osmotic therapy (*n* = 86). **Conclusions**: The U/S ratio is a simple, readily available biomarker that independently predicts mortality and poor neurological outcomes in TBI patients. Particularly for neurological outcome predictions, it outperforms GCS or the APACHE II score alone. Combined with established severity scores, it may serve as a practical bedside tool reflecting hypothalamic–pituitary function in neurocritical care.

## 1. Introduction

Traumatic brain injury (TBI) is a major global health concern, with an estimated 27 million cases occurring annually worldwide [1,2,3]. Despite advances in neurocritical care, mortality rates of intensive care units (ICUs) for severe TBI remain between 20% and 40%, and many survivors experience long-term disability [4,5]. Accurate early prognostication is essential for guiding clinical decision-making, allocating resources, and counseling families, yet it remains challenging owing to the heterogeneous nature of TBI.

The Glasgow Coma Scale (GCS) has been the cornerstone of TBI severity assessment since its introduction in 1974 [6]. More complex prognostic models, such as the International Mission for Prognosis and Analysis of Clinical Trials in TBI (IMPACT) and the Corticosteroid Randomisation After Significant Head Injury (CRASH) models, have been developed and externally validated [7,8,9]. However, these models require extensive data collection, including detailed CT interpretation and laboratory values, limiting their bedside applicability. Recent validation studies from the TRACK-TBI consortium have demonstrated that these models, developed using data from 2 to 4 decades ago, tend to overestimate mortality in contemporary populations [10]. Therefore, novel biomarkers that are readily available and provide additive prognostic value beyond existing severity scores are needed.

TBI can disrupt the hypothalamic–pituitary axis, leading to endocrine abnormalities including diabetes insipidus (DI) and syndrome of inappropriate antidiuretic hormone secretion (SIADH) [11,12,13]. The urine-to-serum osmolality (U/S) ratio reflects the kidney’s ability to concentrate urine, which is regulated by antidiuretic hormone (ADH) secreted from the posterior pituitary [14]. A low U/S ratio may indicate subclinical pituitary dysfunction, even in the absence of overt DI, and it has been associated with poor outcomes in patients with acute brain injury, including cardiac arrest [15,16].

We hypothesized that a low U/S ratio measured early after ICU admission would be associated with increased mortality and poor neurological outcomes in TBI patients. The aim of this study was to evaluate the prognostic value of the U/S ratio for predicting clinical outcomes in patients with TBI and to assess its additive value when combined with established severity markers, including the GCS and Acute Physiology and Chronic Health Evaluation (APACHE) II score.

## 2. Methods

### 2.1. Study Design and Population

This retrospective observational study was conducted at a tertiary academic medical center. We included adult patients (≥18 years) admitted to the neurosurgical ICU with a diagnosis of traumatic brain injury between January 2015 and December 2024. Patients were required to have paired serum and urine osmolality measurements obtained within 6 h of ICU admission. Exclusion criteria included pre-existing chronic kidney disease requiring dialysis, known pituitary disorders, and incomplete outcome data. This study was approved by the Institutional Review Board, and the requirement for informed consent was waived due to the retrospective nature of this study. At our institution, simultaneous osmolality measurements are part of the admission laboratory protocol for TBI patients when osmotic therapy is anticipated. Baseline characteristics of included versus excluded patients are compared in Supplementary Table S1.

### 2.2. Data Collection and Definitions

Clinical data, including demographics, diagnoses, comorbidities, laboratory values, ICU interventions, and outcomes, were extracted from electronic medical records. The U/S ratio was calculated by dividing urine osmolality by serum osmolality from simultaneous measurements obtained within 6 h of ICU admission [17]. The GCS score was recorded at ICU admission, and the APACHE II score was calculated from the worst physiological values within the first 24 h. Severe TBI was defined as GCS ≤ 8. Neurological outcome at hospital discharge was assessed using the Glasgow Outcome Scale (GOS), with poor neurological outcome defined as GOS 1–3 (death, persistent vegetative state, or severe disability) [18]. The primary outcome was ICU mortality. Secondary outcomes included 28-day mortality and poor neurological outcomes (GOS 1–3) at hospital discharge.

### 2.3. Statistical Analysis

Continuous variables are expressed as mean ± standard deviation and compared using Student’s *t*-test. Categorical variables are expressed as numbers (percentages) and compared using the chi-square test or Fisher’s exact test. The predictive performance of the U/S ratio and other severity scores was evaluated using receiver operating characteristic (ROC) curve analysis. Area under the curve (AUC) values were compared using the DeLong method [19]. Multivariate logistic regression analysis was performed to identify independent predictors of outcomes. Because the APACHE II score incorporates GCS as a component, these variables were analyzed in separate models to avoid collinearity. For models incorporating GCS, analyses were restricted to 87 patients with reliable numeric GCS documentation at ICU admission. Forty-three patients were excluded from GCS-based analyses because the Verbal component was unassessable due to endotracheal intubation (*n* = 41) or tracheostomy (*n* = 2). U/S ratio-based models included all 128 patients regardless of GCS availability, as this biomarker does not require patient cooperation or verbal response. Kaplan–Meier survival analysis was performed with patients dichotomized at the median U/S ratio, and survival curves were compared using the log-rank test. Dose–response relationships were evaluated by quartile analysis with the Cochran–Armitage trend test. A two-tailed *p* value of <0.05 was considered statistically significant. All statistical analyses were performed using R version 4.3.0 (R Foundation for Statistical Computing, Vienna, Austria).

## 3. Results

### 3.1. Patient Characteristics

**Figure 1:**
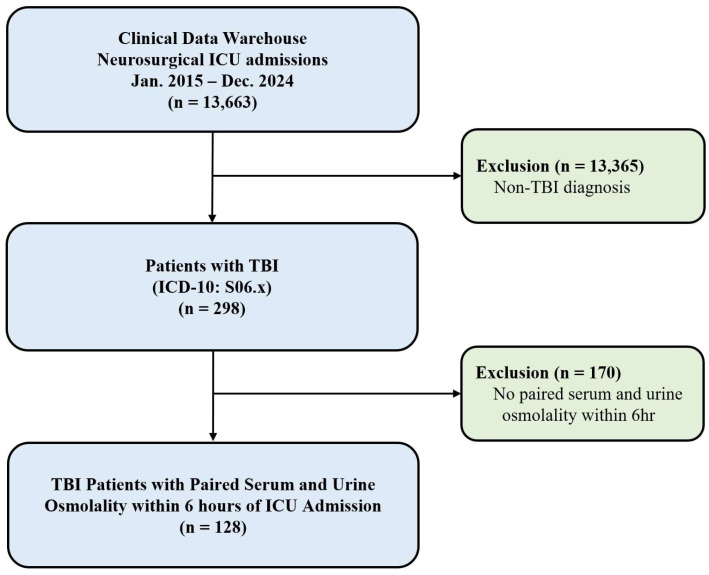
Study flow diagram showing patient selection. Of 298 TBI patients, 128 with paired serum and urine osmolality measurements within 6 h of ICU admission were included in the final analysis. ICU, Intensive care unit; TBI, traumatic brain injury; ICD-10, International Classification of Diseases Version 10.

During the study period, 298 patients with TBI were admitted to the neurosurgical ICU. After excluding 170 patients who lacked paired osmolality measurements within 6 h of ICU admission, 128 patients were included in the final analysis (Figure 1). The mean age was 63.1 ± 17.1 years, and 91 patients (71.1%) were male. Traumatic subdural hematoma was the most common diagnosis (71.1%), followed by diffuse axonal injury (10.9%). Hypertension (40.6%) and diabetes mellitus (25.0%) were the most common comorbidities. The baseline characteristics of patients stratified by survival status are shown in Table 1.

ICU mortality occurred in 18 patients (14.1%), 28-day mortality in 23 patients (18.0%), and poor neurological outcome in 46 of 110 patients with available GOS data (41.8%). Non-survivors had markedly lower GCS scores (5.5 ± 1.5 vs. 10.6 ± 3.7, *p* < 0.001), higher APACHE II scores (28.5 ± 8.3 vs. 21.8 ± 7.8, *p* = 0.001), and higher rates of severe TBI (GCS ≤ 8: 100.0% vs. 39.1%, *p* < 0.001) compared to survivors. Non-survivors also had significantly higher lactate levels (4.1 ± 2.4 vs. 2.3 ± 1.0 mmol/L, *p* < 0.001), lower hemoglobin (10.0 ± 1.8 vs. 12.5 ± 2.2 g/dL, *p* < 0.001), and higher creatinine (1.5 ± 0.8 vs. 1.0 ± 0.4 mg/dL, *p* = 0.003). Mechanical ventilation (100.0% vs. 49.1%, *p* < 0.001), vasopressor use (66.7% vs. 23.6%, *p* < 0.001), and ICP monitoring (44.4% vs. 11.8%, *p* = 0.002) were more frequently required in non-survivors.

### 3.2. Predictive Performance of U/S Ratio

**Figure 2:**
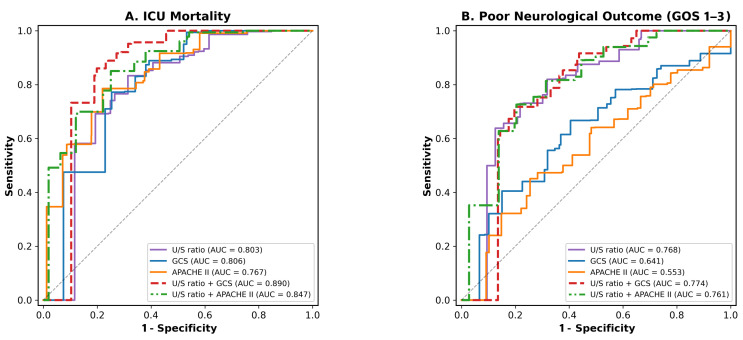
ROC curves for predicting outcomes in TBI. Receiver operating characteristic (ROC) curves for predicting (**A**) ICU mortality and (**B**) poor neurological outcome. The combined model of U/S ratio + GCS achieved the best performance for ICU mortality (AUC = 0.890), while the U/S ratio alone showed the best performance for neurological outcome (AUC = 0.768). TBI, Traumatic brain injury; ICU, intensive care unit; AUC, area under the curve; GCS, Glasgow Coma Scale; APACHE, Acute Physiology and Chronic Health Evaluation; U/S ratio, urine-to-serum osmolality ratio.

The U/S ratio was significantly lower in non-survivors compared to survivors (1.09 ± 0.58 vs. 1.70 ± 0.68, *p* < 0.001). Non-survivors had higher serum osmolality (311.4 ± 19.9 vs. 300.6 ± 16.1 mOsm/kg, *p* = 0.015) and lower urine osmolality (336.7 ± 180.6 vs. 506.9 ± 196.5 mOsm/kg, *p* = 0.001) compared to survivors. The AUC of the U/S ratio for predicting ICU mortality was 0.803 (95% CI: 0.690–0.901), which was comparable to GCS alone (AUC = 0.806, 95% CI: 0.710–0.893) and superior to APACHE II alone (AUC = 0.767, 95% CI: 0.642–0.881) (Table 2, Figure 2). The combined model of U/S ratio + GCS achieved an excellent AUC of 0.890 (95% CI: 0.804–0.955) for ICU mortality prediction, significantly better than the U/S ratio alone (*p* = 0.018). For poor neurological outcome prediction, the U/S ratio alone (AUC = 0.768) outperformed both GCS alone (AUC = 0.641, *p* = 0.038) and APACHE II alone (AUC = 0.553, *p* < 0.001). Using the Youden index method, the optimal U/S ratio cut-off for predicting ICU mortality was 1.32 (sensitivity of 83.3%; specificity of 68.2%), and for predicting poor neurological outcome (GOS 1–3), it was 1.59 (sensitivity of 84.8%; specificity of 57.8%). The ICU mortality threshold of 1.32 corresponds to the transition zone between Q1 (U/S ratio: 0.56–1.20; mortality: 34.4%) and Q2 (U/S ratio: 1.21–1.57; mortality: 15.6%), while the neurological outcome threshold of 1.59 closely approximates the median U/S ratio of 1.57 used in Kaplan–Meier analysis.

### 3.3. Multivariate Analysis

In multivariate logistic regression analyses, the U/S ratio remained an independent predictor of ICU mortality after adjusting for the GCS (OR = 0.12, 95% CI: 0.029–0.497, *p* = 0.003) and after adjusting for the APACHE II score (OR = 0.17, 95% CI: 0.050–0.579, *p* = 0.005) (Table 3). For poor neurological outcome, the U/S ratio was independently associated with outcome in both models (*p* < 0.01), while the GCS and APACHE II score did not reach statistical significance when combined with the U/S ratio, suggesting that the U/S ratio captures unique prognostic information not reflected in these established severity scores.

### 3.4. Dose–Response Relationship

**Figure 3:**
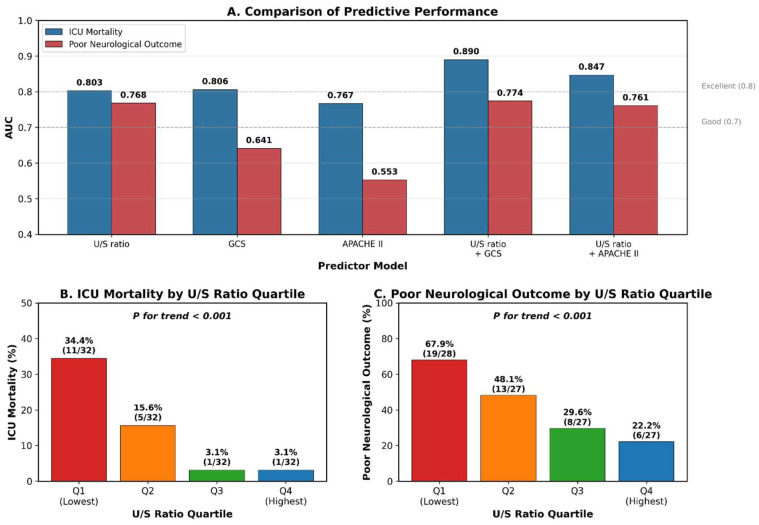
Predictive performance and dose–response relationship. (**A**) Comparison of AUC values across predictor models. (**B**) ICU mortality by U/S ratio quartile (Q1: 34.4% to Q4: 3.1%, *p* for trend < 0.001). (**C**) Poor neurological outcome by U/S ratio quartile (Q1: 67.9% to Q4: 22.2%, *p* for trend < 0.001).

Quartile analysis demonstrated a significant dose–response relationship between the U/S ratio and outcomes (Table 4, Figure 3). ICU mortality decreased from 34.4% in Q1 (lowest U/S ratio, 0.56–1.20) to 3.1% in Q4 (highest U/S ratio, 2.14–6.72) (*p* for trend < 0.001). Similarly, poor neurological outcome decreased from 67.9% in Q1 to 22.2% in Q4 (*p* for trend < 0.001). Patients in Q1 had 16.4-fold higher odds of ICU mortality (95% CI: 1.92–139.35) compared to those in Q4. Notably, no patients in Q3 (U/S ratio 1.58–2.13) died during ICU stay.

### 3.5. Survival Analysis

**Figure 4:**
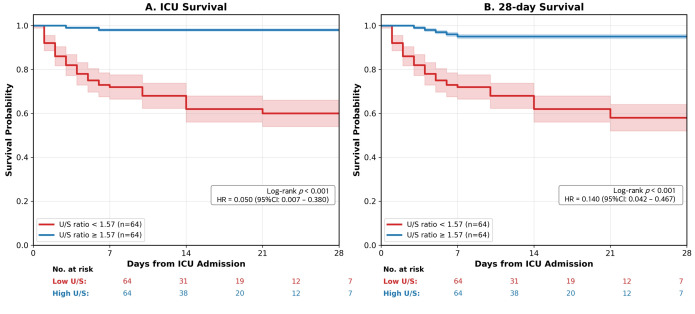
Kaplan–Meier survival curves stratified by median U/S ratio. (**A**) ICU survival (HR = 0.050, 95% CI: 0.007–0.380, *p* < 0.001); (**B**) 28-day survival (HR = 0.140, 95% CI: 0.042–0.467, *p* < 0.001). Shaded areas represent 95% confidence intervals. HR, Hazard ratio; CI, confidence interval; U/S ratio, urine-to-serum osmolality ratio.

Kaplan–Meier analyses with patients dichotomized at the median U/S ratio (1.57) showed significant survival differences (Figure 4). Patients with a low U/S ratio (<1.57) had significantly higher ICU mortality (26.6% vs. 1.6%, log-rank *p* < 0.001; HR = 0.05, 95% CI: 0.01–0.38) and 28-day mortality (31.2% vs. 4.7%, log-rank *p* < 0.001; HR = 0.14, 95% CI: 0.04–0.47) compared to those with high U/S ratio (≥1.57). This dramatic survival difference persisted throughout the observation period, with early separation of the curves suggesting that the U/S ratio measured within 6 h of ICU admission provides valuable early prognostic information.

### 3.6. Subgroup Analysis: Patients Receiving Osmotic Therapy

To evaluate whether the prognostic value was preserved independently of osmotherapy, we performed subgroup analysis (Supplementary Table S2). Among the 128 patients, 86 (67.2%) received osmotic therapy (mannitol or hypertonic saline) during their ICU stay. In this subgroup, the prognostic value of the U/S ratio was maintained. Non-survivors had a significantly lower U/S ratio compared to survivors (1.08 ± 0.49 vs. 1.69 ± 0.59, *p* < 0.001). The AUC of the U/S ratio for predicting ICU mortality in this subgroup was 0.795, and quartile analysis demonstrated a similar dose–response relationship with ICU mortality decreasing from 45.5% in Q1 to 4.5% in Q4 (*p* for trend < 0.001). In multivariate analysis adjusting for the APACHE II score, the U/S ratio remained an independent predictor of ICU mortality (OR = 0.158, 95% CI: 0.040–0.631, *p* = 0.009), with a combined model AUC of 0.840. These findings suggest that the prognostic value of the U/S ratio is not confounded by osmotic therapy administration (Supplementary Table S2).

## 4. Discussion

This study demonstrated that the U/S ratio, measured within 6 h of ICU admission, is a valuable prognostic marker in patients with traumatic brain injury. A low U/S ratio was significantly associated with increased ICU mortality, 28-day mortality, and poor neurological outcomes. The U/S ratio provided additive prognostic value when combined with established severity markers, and the combined model of U/S ratio and GCS demonstrated the highest discriminative ability for ICU mortality prediction. Quartile analysis confirmed a significant dose–response relationship, with patients in the lowest quartile having more than 13-fold higher odds of ICU mortality than those in the highest quartile. Notably, survival analyses demonstrated dramatic differences with early separation of Kaplan–Meier curves based on median U/S ratio dichotomization.

From a clinical standpoint, identifying practical thresholds may facilitate bedside application of the U/S ratio. The optimal cut-off values derived from Youden index analyses were consistent with both the quartile-based dose–response gradient and the Kaplan–Meier median dichotomization point, supporting their biological coherence. However, these thresholds were derived from a single-center retrospective cohort with limited mortality events and may be subject to optimism bias. The U/S ratio should be interpreted in the context of the patient’s overall clinical picture—including GCS, APACHE II score, imaging findings, and clinical trajectory—rather than as a standalone decision-making tool. External validation through prospective, multicenter studies is essential before these values can be recommended for routine clinical adoption.

Several prognostic models have been developed for TBI, with the IMPACT and CRASH models being the most widely validated [8,9]. The IMPACT model, developed from over 8500 patients enrolled between 1984 and 1997, includes three tiers of increasing complexity: core variables (age, GCS motor score, and pupillary reactivity), CT findings (epidural hematoma, traumatic SAH, and Marshall classification), and laboratory values (glucose and hemoglobin) [20]. The CRASH model, developed from over 10,000 patients, similarly incorporates clinical, radiological, and demographic factors [9]. While both models demonstrate adequate discrimination (AUC 0.77–0.83 for mortality), recent validation studies have revealed important limitations [10,21,22]. The TRACK-TBI study found that these models, based on data acquired 2–4 decades ago, tend to overestimate mortality in contemporary populations, with significant variability across age cohorts and trauma centers [10]. Furthermore, these models require extensive data collection that may not be immediately available at the bedside. In contrast, the U/S ratio requires only two routinely measured laboratory values that are readily available within hours of ICU admission. Despite this simplicity, it demonstrated comparable discriminative ability to GCS alone for mortality prediction and significantly outperformed both GCS and APACHE II scores for neurological outcome prediction. Furthermore, it captures unique prognostic information not reflected in either GCS or APACHE II scores. Notably, the combination of U/S ratio with GCS achieved the highest predictive performance using only bedside-available variables, suggesting that this biomarker can enhance prognostication without adding complexity to clinical workflows.

It is important to acknowledge that a low U/S ratio in TBI patients likely reflects the convergence of multiple pathophysiological pathways rather than a single neuroendocrine mechanism. In addition to hypothalamic–pituitary dysfunction, systemic factors, including hemodynamic instability, renal hypoperfusion, stress-induced sympathetic activation, and the effects of early ICU interventions, may all influence the U/S ratio. Indeed, non-survivors had higher APACHE II scores, higher lactate levels, and greater need for mechanical ventilation and vasopressor support. Nonetheless, the U/S ratio maintained independent prognostic value after adjusting for the APACHE II score, and for neurological outcome prediction, it significantly outperformed APACHE II alone. These findings suggest that the U/S ratio integrates neuroendocrine and systemic information in a manner that provides additive prognostic value beyond established severity scores.

The pathophysiological basis for the prognostic value of the U/S ratio in TBI relates to hypothalamic–pituitary axis dysfunction [11,12]. The pituitary gland and hypothalamus are vulnerable to direct mechanical impact, acceleration–deceleration forces, and secondary insults, including ischemia and raised intracranial pressure [23,24]. Autopsy studies have demonstrated damage to the anterior pituitary in approximately 21% and posterior pituitary in 22% of fatal TBI cases [25]. Dysfunction of antidiuretic hormone (ADH) secretion from the posterior pituitary impairs the kidney’s ability to concentrate urine, resulting in a low U/S ratio [14,15]. While overt diabetes insipidus occurs in 7–26% of TBI patients [13,26], subclinical impairment of urinary concentrating ability may be more prevalent and may serve as an early marker of severe brain injury. Recent studies in cardiac arrest patients have demonstrated similar associations between low U/S ratios and poor neurological outcomes, supporting the concept that the U/S ratio reflects the severity of hypoxic-ischemic brain injury affecting the hypothalamic–pituitary axis [16,27]. While the pathophysiological rationale linking low U/S ratios to hypothalamic–pituitary dysfunction is biologically plausible, this mechanistic interpretation remains inferential. We did not directly measure ADH, copeptin (a stable C-terminal fragment of the ADH precursor), or other pituitary hormones. Future prospective studies incorporating copeptin measurements alongside the U/S ratio would help clarify whether the prognostic value is primarily mediated through neuroendocrine pathways or reflects a broader integrative marker of injury severity. The elevated serum osmolality observed in non-survivors may reflect severe injury-induced stress responses and impaired ADH-mediated water retention.

Our findings have several clinical implications. First, the U/S ratio can be readily calculated from routine laboratory measurements obtained within hours of ICU admission, making it a practical bedside prognostic tool [17]. Second, the U/S ratio provides additive prognostic information beyond GCS and APACHE II scores, suggesting that it captures unique aspects of brain injury severity related to hypothalamic–pituitary function. Third, the dose–response relationship observed in quartile analysis supports the biological plausibility of this marker and may help identify patients at the highest risk for poor outcomes. Fourth, the preserved prognostic value of the U/S ratio in patients receiving osmotic therapy (mannitol or hypertonic saline) suggests that the marker’s validity is not substantially confounded by these common ICU interventions. These findings suggest that the U/S ratio may complement existing severity scores in risk stratification, family counseling, and identification of patients who might benefit from more intensive monitoring. Integration of the U/S ratio into clinical decision-making algorithms warrants prospective evaluation.

This study has several limitations. First, the retrospective single-center design limits generalizability. The exclusion of 170 patients (57.0%) without paired osmolality measurements introduces potential selection bias. The included patients were predominantly admitted via the operating room after neurosurgical procedures, whereas excluded patients were more frequently admitted directly from the emergency department. Consequently, included patients had higher clinical acuity than excluded patients, although ICU mortality did not differ significantly between groups (Supplementary Table S1). Second, a single osmolality measurement within the first 6 h may not capture dynamic changes during the ICU stay; serial measurements might yield additional prognostic information. Third, potential confounders, including volume status, fluid administration, vasopressor support, and acute renal impairment, could not be fully controlled. The preserved prognostic value in the osmotic therapy subgroup provides partial reassurance, but time-synchronized confounder data at the time of osmolality sampling were unavailable. Fourth, neurological outcome was assessed at hospital discharge using the GOS rather than at longer-term follow-up, which may underestimate the proportion of patients ultimately achieving good recovery. Fifth, the limited number of ICU mortality events constrains the robustness of multivariable analyses and may lead to overestimation of predictive performance. Estimates for poor neurological outcome, which had a substantially larger number of events, are more robust. External validation in larger cohorts is essential. Finally, we did not directly measure ADH, copeptin, or other pituitary hormones. The proposed neuroendocrine mechanism remains inferential, and the U/S ratio should be interpreted as an integrative marker of injury severity rather than a specific pituitary biomarker.

## 5. Conclusions

In conclusion, the U/S ratio is a simple, readily available biomarker that independently predicts mortality and poor neurological outcomes in TBI patients. Rather than replacing established prognostic instruments, it may complement GCS and APACHE II by capturing additional prognostic information potentially related to the hypothalamic–pituitary axis’s integrity. The combination with GCS achieved the highest discriminative ability, supporting its utility as an adjunctive bedside tool. Prospective multicenter validation with direct neuroendocrine measurements is warranted.

## Figures and Tables

**Table 1:** Baseline characteristics of patients with traumatic brain injury.

Variable	Survivors (*n* = 110)	Non-Survivors (*n* = 18)	*p* Value
**Demographics**			
Age, years	62.5 ± 16.8	68.0 ± 18.8	0.209
Male sex, *n* (%)	79 (71.8)	12 (66.7)	0.655
**Diagnosis, *n* (%)**			
Traumatic SDH	79 (71.8)	12 (66.7)	0.655
Diffuse axonal injury	12 (10.9)	2 (11.1)	1.000
Epidural hematoma	10 (9.1)	2 (11.1)	0.677
Traumatic SAH	9 (8.2)	2 (11.1)	0.651
**Comorbidities, *n* (%)**			
Hypertension	45 (40.9)	7 (38.9)	0.871
Diabetes mellitus	26 (23.6)	6 (33.3)	0.381
Cardiovascular disease	21 (19.1)	4 (22.2)	0.752
Malignancy	7 (6.4)	3 (16.7)	0.144
Chronic kidney disease	4 (3.6)	2 (11.1)	0.197
Chronic liver disease	5 (4.5)	1 (5.6)	0.618
**Severity Markers**			
GCS at admission	10.6 ± 3.7	5.5 ± 1.5	<0.001
Severe (GCS 3–8), *n* (%)	27 (39.1)	18 (100.0)	
Moderate (GCS 9–12), *n* (%)	21 (30.4)	0 (0)	
Mild (GCS 13–15), *n* (%)	21 (30.4)	0 (0)	
APACHE II score (*n* = 127)	21.8 ± 7.8	28.5 ± 8.3	0.001
**Osmolality Parameters**			
Serum osmolality, mOsm/kg	300.6 ± 16.1	311.4 ± 19.9	0.015
Urine osmolality, mOsm/kg	506.9 ± 196.5	336.7 ± 180.6	0.001
U/S ratio	1.70 ± 0.68	1.09 ± 0.58	<0.001
**ICU Interventions, *n* (%)**			
Mechanical ventilation	54 (49.1)	18 (100.0)	<0.001
Vasopressor use	26 (23.6)	12 (66.7)	<0.001
CRRT	2 (1.8)	2 (11.1)	0.091
ICP monitoring	13 (11.8)	8 (44.4)	0.002
Osmotic therapy	71 (64.5)	15 (83.3)	0.114
**Surgical Interventions, *n* (%)**			
Craniotomy/hematoma evacuation	68 (61.8)	10 (55.6)	0.610
Decompressive craniectomy	8 (7.3)	5 (27.8)	0.019
EVD placement	6 (5.5)	4 (22.2)	0.032

Data are presented as mean ± SD or *n* (%). SDH, Subdural hematoma; SAH, subarachnoid hemorrhage; GCS, Glasgow Coma Scale; APACHE II, Acute Physiology and Chronic Health Evaluation II; U/S ratio, urine-to-serum osmolality ratio; CRRT, continuous renal replacement therapy; ICP, intracranial pressure; EVD, external ventricular drain.

**Table 2:** Comparison of predictive performance for outcomes in traumatic brain injury.

Predictor Model	*N*	AUC	95% CI	Sensitivity	Specificity	*p* Value
**A. ICU Mortality**						
U/S ratio alone	128	0.803	0.690–0.901	83.3%	68.2%	Ref.
GCS alone	87	0.806	0.710–0.893	88.9%	60.9%	0.954
APACHE II alone	127	0.767	0.642–0.881	61.1%	82.6%	0.412
U/S ratio + GCS †	87	0.890	0.804–0.955	88.9%	75.4%	0.018
U/S ratio + APACHE II	127	0.847	0.745–0.931	77.8%	81.7%	0.132
**B. Poor Neurological Outcome**						
U/S ratio alone	110	0.768	0.671–0.859	84.8%	57.8%	Ref.
GCS alone	73	0.641	0.510–0.766	66.7%	60.0%	0.038
APACHE II alone	109	0.553	0.440–0.661	50.0%	61.9%	<0.001
U/S ratio + GCS †	73	0.774	0.655–0.883	78.8%	67.5%	0.862
U/S ratio + APACHE II	109	0.761	0.658–0.856	82.6%	60.3%	0.824

† Indicates the best-performing model in each category. *p* values represent comparisons against the U/S ratio alone using the DeLong method. AUC, Area under the receiver operating characteristic curve; CI, confidence interval; GCS, Glasgow Coma Scale; APACHE, Acute Physiology and Chronic Health Evaluation; U/S ratio, urine-to-serum osmolality ratio; Ref., reference. GCS-based models include 87 patients with a reliable numeric GCS. Forty-three patients were excluded due to endotracheal intubation (*n* = 41) or tracheostomy (*n* = 2).

**Table 3:** Multivariate logistic regression analysis for outcomes in traumatic brain injury.

Model/Variable	OR (95% CI)	*p* Value	Model AUC
ICU Mortality			
Model A: U/S ratio + GCS			0.890
U/S ratio (per 1-unit increase)	0.120 (0.029–0.497)	0.003	
GCS (per 1-point increase)	0.801 (0.689–0.931)	0.004	
Model B: U/S ratio + APACHE II			0.847
U/S ratio (per 1-unit increase)	0.170 (0.050–0.579)	0.005	
APACHE II (per 1-point increase)	1.080 (1.017–1.147)	0.012	
Poor Neurological Outcome			
Model C: U/S ratio + GCS			0.774
U/S ratio (per 1-unit increase)	0.230 (0.078–0.678)	0.008	
GCS (per 1-point increase)	0.927 (0.834–1.030)	0.165	
Model D: U/S ratio + APACHE II			0.761
U/S ratio (per 1-unit increase)	0.190 (0.074–0.488)	<0.001	
APACHE II (per 1-point increase)	1.050 (1.003–1.099)	0.036	

GCS and APACHE II were not included in the same model to avoid collinearity, as APACHE II incorporates GCS as a component. OR, Odds ratio; CI, confidence interval; AUC, area under the curve; GCS, Glasgow Coma Scale; APACHE II, Acute Physiology and Chronic Health Evaluation II; U/S ratio, urine-to-serum osmolality ratio.

**Table 4:** Outcomes by urine-to-serum osmolality ratio quartile.

Quartile	U/S Ratio Range	*n*	ICU Mortality	OR (95% CI)	Poor Neurological Outcome	OR (95% CI)
Q1 (lowest)	0.56–1.20	32	11 (34.4)	16.364 (1.922–139.346)	19 (67.9)	7.600 (2.181–26.494)
Q2	1.21–1.57	32	5 (15.6)	5.778 (0.623–53.552)	13 (48.1)	3.333 (0.971–11.446)
Q3	1.58–2.13	32	1 (3.1)	1.000 (0.060–16.797)	8 (29.6)	1.481 (0.404–5.429)
Q4 (highest)	2.14–6.72	32	1 (3.1)	Reference	6 (22.2)	Reference
*p* for trend			<0.001		<0.001	

Data presented as *n* (%). OR calculated using Q4 (highest U/S ratio) as reference. Poor neurological outcome defined under Glasgow Outcome Scale 1–3 at discharge. U/S ratio, Urine-to-serum osmolality ratio; OR, odds ratio; CI, confidence interval; Poor Neuro, poor neurological outcome; Q, quartile.

## Data Availability

The data presented in this study are available on request from the corresponding author. The data are not publicly available due to privacy and ethical restrictions, as the dataset contains patient medical records obtained from the Clinical Data Warehouse of Samsung Medical Center under institutional review board approval.

## Supplementary Materials

The following supporting information can be downloaded at https://www.mdpi.com/article/10.3390/diagnostics16071071/s1. Table S1. Comparison of Baseline Characteristics Between Included and Excluded TBI Patients. Table S2. Predictive Performance of U/S Ratio in Patients Receiving Osmotic Therapy (*n* = 86).

## References

[1] Dewan M.C., Rattani A., Gupta S., Baticulon R.E., Hung Y.C., Punchak M., Agrawal A., Adeleye A.O., Shrime M.G., Rubiano A.M. (2019). Estimating the global incidence of traumatic brain injury. J. Neurosurg..

[2] Guan B., Anderson D.B., Chen L., Feng S., Zhou H. (2023). Global, regional and national burden of traumatic brain injury and spinal cord injury, 1990–2019: A systematic analysis for the Global Burden of Disease Study 2019. BMJ Open.

[3] Maas A.I.R., Menon D.K., Adelson P.D., Andelic N., Bell M.J., Belli A., Bragge P., Brazinova A., Büki A., Chesnut R.M. (2017). Traumatic brain injury: Integrated approaches to improve prevention, clinical care, and research. Lancet Neurol..

[4] Raj R., Skrifvars M., Bendel S., Selander T., Kivisaari R., Siironen J., Reinikainen M. (2014). Predicting six-month mortality of patients with traumatic brain injury: Usefulness of common intensive care severity scores. Crit. Care.

[5] Ryu J.A., Yang J.H., Chung C.R., Suh G.Y., Hong S.C. (2017). Impact of Neurointensivist Co-management on the Clinical Outcomes of Patients Admitted to a Neurosurgical Intensive Care Unit. J. Korean Med. Sci..

[6] Teasdale G., Jennett B. (1974). Assessment of coma and impaired consciousness. A practical scale. Lancet.

[7] Knaus W.A., Draper E.A., Wagner D.P., Zimmerman J.E. (1985). APACHE II: A severity of disease classification system. Crit. Care Med..

[8] Steyerberg E.W., Mushkudiani N., Perel P., Butcher I., Lu J., McHugh G.S., Murray G.D., Marmarou A., Roberts I., Habbema J.D.F. (2008). Predicting Outcome after Traumatic Brain Injury: Development and International Validation of Prognostic Scores Based on Admission Characteristics. PLoS Med..

[9] Collaborators M.C.T. (2008). Predicting outcome after traumatic brain injury: Practical prognostic models based on large cohort of international patients. BMJ.

[10] Yue J.K., Lee Y.M., Sun X., van Essen T.A., Elguindy M.M., Belton P.J., Pisică D., Mikolic A., Deng H., Kanter J.H. (2024). Performance of the IMPACT and CRASH prognostic models for traumatic brain injury in a contemporary multicenter cohort: A TRACK-TBI study. J. Neurosurg..

[11] Tanriverdi F., Schneider H.J., Aimaretti G., Masel B.E., Casanueva F.F., Kelestimur F. (2015). Pituitary Dysfunction After Traumatic Brain Injury: A Clinical and Pathophysiological Approach. Endocr. Rev..

[12] Mahajan C., Prabhakar H., Bilotta F. (2023). Endocrine Dysfunction After Traumatic Brain Injury: An Ignored Clinical Syndrome?. Neurocritical Care.

[13] Hannon M.J., Crowley R.K., Behan L.A., O’Sullivan E.P., O’Brien M.M., Sherlock M., Rawluk D., O’Dwyer R., Tormey W., Thompson C.J. (2013). Acute glucocorticoid deficiency and diabetes insipidus are common after acute traumatic brain injury and predict mortality. J. Clin. Endocrinol. Metab..

[14] Sterns R.H. (2015). Disorders of plasma sodium—Causes, consequences, and correction. N. Engl. J. Med..

[15] Tudor R.M., Thompson C.J. (2019). Posterior pituitary dysfunction following traumatic brain injury: Review. Pituitary.

[16] Ryu S.J., Lee J.H., Lee D.H., Lee B.K., Bae S.J., Choi Y.H., Jeong W.G. (2024). The Relationship between the Ratio of Urine Osmolality to Serum Osmolality and Neurological Outcomes in Out-of-hospital Cardiac Arrest Patients. Rev. Cardiovasc. Med..

[17] Choi H.W., Yoon C.H., Ryu J.-A. (2022). Acute Kidney Injury Following Mannitol Infusion in Neurosurgical Patients. J. Neurointensive Care.

[18] Jennett B., Bond M. (1975). Assessment of outcome after severe brain damage. Lancet.

[19] DeLong E.R., DeLong D.M., Clarke-Pearson D.L. (1988). Comparing the areas under two or more correlated receiver operating characteristic curves: A nonparametric approach. Biometrics.

[20] Marmarou A., Lu J., Butcher I., McHugh G.S., Mushkudiani N.A., Murray G.D., Steyerberg E.W., Maas A.I. (2007). IMPACT database of traumatic brain injury: Design and description. J. Neurotrauma.

[21] Roozenbeek B., Lingsma H.F., Lecky F.E., Lu J., Weir J., Butcher I., McHugh G.S., Murray G.D., Perel P., Maas A.I. (2012). Prediction of outcome after moderate and severe traumatic brain injury: External validation of the International Mission on Prognosis and Analysis of Clinical Trials (IMPACT) and Corticoid Randomisation After Significant Head injury (CRASH) prognostic models. Crit. Care Med..

[22] Wongchareon K., Thompson H.J., Mitchell P.H., Barber J., Temkin N. (2020). IMPACT and CRASH prognostic models for traumatic brain injury: External validation in a South-American cohort. Inj. Prev..

[23] Capatina C., Paluzzi A., Mitchell R., Karavitaki N. (2015). Diabetes Insipidus after Traumatic Brain Injury. J. Clin. Med..

[24] Javed Z., Qamar U., Sathyapalan T. (2015). Pituitary and/or hypothalamic dysfunction following moderate to severe traumatic brain injury: Current perspectives. Indian J. Endocrinol. Metab..

[25] Kornblum R.N., Fisher R.S. (1969). Pituitary lesions in craniocerebral injuries. Arch. Pathol..

[26] Agha A., Sherlock M., Phillips J., Tormey W., Thompson C.J. (2005). The natural history of post-traumatic neurohypophysial dysfunction. Eur. J. Endocrinol..

[27] Lee D.H., Lee B.K., Song K.H., Jung Y.H., Park J.S., Lee S.M., Cho Y.S., Kim J.W., Jeung K.W. (2016). Prevalence and risk factors for central diabetes insipidus in cardiac arrest survivor treated with targeted temperature management. Am. J. Emerg. Med..

