# Supplementary material for: Urine-to-Serum Osmolality Ratio as a Prognostic Marker in Traumatic Brain Injury
Source: Diagnostics (Basel). 2026 Apr 2;16(7):1071. doi: 10.3390/diagnostics16071071 (PMC13072897; doi:10.3390/diagnostics16071071)
Supplement: Supplementary file 1 [file diagnostics-16-01071-s001.zip › diagnostics-4165797-supplementary.pdf]

Supplementary Materials

Urine-to-Serum Osmolality Ratio as a Prognostic Marker in Traumatic Brain Injury

Eun Young Kim, MD and Jeong-Am Ryu, MD, PhD

Diagnostics 2026, Manuscript ID: diagnostics-4165797

Table S1. Comparison of Baseline Characteristics Between Included and Excluded TBI Patients

Variable	Included (n=128)	Excluded (n=182)	P value
**Demographics**			
Age, years	63.3 ± 17.0	63.8 ± 18.5	0.801
Male sex, n (%)	91 (71.1)	122 (67.0)	0.408
**Severity Markers**			
GCS at admission	10.5 ± 4.4	11.6 ± 3.9	0.021
Severe TBI (GCS ≤8), n (%)	43 (33.6)	41 (22.5)	0.042
Moderate TBI (GCS 9–12), n (%)	26 (20.3)	32 (17.6)	
Mild TBI (GCS 13–15), n (%)	59 (46.1)	109 (59.9)	
APACHE II score	23.0 ± 8.5	19.2 ± 8.0	<0.001
**Diagnosis, n (%)**			
Traumatic SDH	91 (71.1)	96 (52.7)	
Diffuse brain injury	14 (10.9)	29 (15.9)	
Epidural hemorrhage	12 (9.4)	16 (8.8)	
Traumatic SAH	11 (8.6)	22 (12.1)	
**ICU Admission Pathway, n (%)**			
ICU admission via OR	84 (65.6)	15 (8.2)	<0.001
ICU admission via ER	30 (23.4)	123 (67.6)	
**Interventions, n (%)**			
Mechanical ventilation	75 (58.6)	74 (40.7)	0.003
CRRT	4 (3.1)	10 (5.5)	0.468
**Outcomes**			
ICU mortality, n (%)	18 (14.1)	18 (9.9)	0.343
ICU length of stay, days	4.8 ± 5.5	4.4 ± 6.2	0.629

Data are presented as mean ± SD or n (%). Included patients had simultaneous serum and urine osmolality measurements within 6 hours of ICU admission. Excluded patients lacked paired osmolality measurements within this time window. The excluded group was identified through comprehensive Clinical Data Warehouse screening using ICD-10 codes (S06.x), which yielded a slightly larger comparator pool (n = 182) than the study flow diagram denominator (n = 170) due to broader diagnostic code screening criteria applied for the selection bias assessment.

Abbreviations: TBI, traumatic brain injury; GCS, Glasgow Coma Scale; APACHE II, Acute Physiology and Chronic Health Evaluation II; SDH, subdural hematoma; SAH, subarachnoid hemorrhage; OR, operating room; ER, emergency room; CRRT, continuous renal replacement therapy; ICU, intensive care unit.

Table S2. Predictive Performance of U/S Ratio in Patients Receiving Osmotic Therapy (n = 86)

Variable	Survivors (n=71)	Non-survivors (n=15)	P value
**Osmolality Parameters**			
Serum osmolality, mOsm/kg	302.4 ± 16.8	314.2 ± 20.5	0.018
Urine osmolality, mOsm/kg	512.3 ± 188.4	342.1 ± 165.8	<0.001
U/S ratio	1.69 ± 0.59	1.08 ± 0.49	<0.001
**ROC Analysis for ICU Mortality**			
U/S ratio alone, AUC (95% CI)		0.795 (0.668–0.902)	
Sensitivity / Specificity		80.0% / 67.6%	
U/S ratio + APACHE II, AUC (95% CI)		0.840 (0.721–0.936)	
**Multivariate Analysis (U/S ratio + APACHE II)**			
U/S ratio, OR (95% CI)	0.158 (0.040–0.631)		0.009
APACHE II, OR (95% CI)	1.085 (1.012–1.163)		0.021
**Quartile Analysis: ICU Mortality, n (%)**			
Q1 (U/S ratio 0.56–1.15)		10/22 (45.5)	
Q2 (U/S ratio 1.16–1.55)		3/21 (14.3)	
Q3 (U/S ratio 1.56–2.05)		1/21 (4.8)	
Q4 (U/S ratio 2.06–6.72)		1/22 (4.5)	
P for trend		<0.001	

Data are presented as mean ± SD or n (%). Osmotic therapy includes mannitol and/or hypertonic saline administration during ICU stay.

Abbreviations: U/S ratio, urine-to-serum osmolality ratio; AUC, area under the curve; CI, confidence interval; OR, odds ratio; APACHE II, Acute Physiology and Chronic Health Evaluation II; Q, quartile; ICU, intensive care unit; ROC, receiver operating characteristic.
